# Cryptic t(15;17) acute promyelocytic leukemia with a karyotype of add(11)(p15) and t(13;20) – A case report with a literature review

**DOI:** 10.17305/bjbms.2020.5106

**Published:** 2021-04

**Authors:** Siyu Gu, Jie Zi, Jinlong Ma, Zheng Ge

**Affiliations:** Department of Hematology, Zhongda Hospital, School of Medicine, Southeast University, Institute of Hematology Southeast University, Nanjing, China

**Keywords:** Cryptic t(15;17), acute promyelocytic leukemia, complex karyotype, *PML/RARα* fusion

## Abstract

Most acute promyelocytic leukemias (APL) are characterized by reciprocal translocations t(15;17)(q22;21), which results in the fusion of the promyelocytic leukemia protein (*PML*) gene at 15q22 with retinoic acid receptor a (*RARα*) gene at 17q21. However, several complex variant translocations also have been reported. Here, we report a 62-year-old man with typical morphology and clinical features of APL with a complex karyotype including add(11)(p15) and t(13;20)(q12;q11.2) without typical t(15;17) assayed by the G-banding analysis. The fluorescence in situ hybridization with a *PML/RARα* dual-color DNA probe showed an atypical fusion signal, quantitative real-time polymerase chain reaction analysis showed *PML/RARα* fusion transcripts, and NGS detected *FLT3*, *WT1*, and *KRAS* mutations. The patient achieved complete remission after treatment with conventional chemotherapy combined with all-trans retinoic acid (ATRA) and arsenic trioxide (ATO). Although the mechanism of this kind of cryptic variant remains unknown, we conclude that the cryptic *PML/RARα* fusion with add(11)(p15) and t(13;20)(q12;q11.2) seems not to alter the effectiveness of chemotherapy combined with ATRA and ATO.

## INTRODUCTION

Acute promyelocytic leukemia (APL) is classified as M3 subtype of acute myeloid leukemia (AML) according to French–American–British (FAB) classification systems, which accounts for approximately 4% to 20% of adult AML cases [1-3]. Typical APL is caused by the t(15;17)(q24.1;q21.2) translocation which leads to a promyelocytic leukemia protein (*PML*) gene and retinoic acid receptor a (*RARα*) gene rearrangement on der(15)and a *RARα/PML* rearrangement on der(17) at the molecular level. *PML/RARα* and *RARα/PML* were found in, respectively, 100% and 67% of patients with positive t(15;17). The PML/RARa fusion protein maintains the ligand-binding domain of RARa and the functional domain of PML, which obstructs the transcription of genes to drive leukemogenesis by deregulating differentiation and self-renewal of myeloid progenitors [4].

In some APL cases, the classic translocation is undetectable, which is considered as masked or cryptic t(15;17). These cases have been detected as submicroscopic insertions of *PML* or *RARα* or as more complex rearrangements such as three- or four-way translocations [5-8]. Here, we report a patient with typical morphology and clinical features of APL with a complex karyotype including add(11)(p15) and t(13;20)(q12;q11.2) but masked t(15;17) based on the conventional chromosome (CC) analysis. The patient achieved complete remission with induction treatment of chemotherapy combined with all-trans retinoic acid (ATRA) and arsenic trioxide (ATO).

## CASE REPORT

**FIGURE 1:**
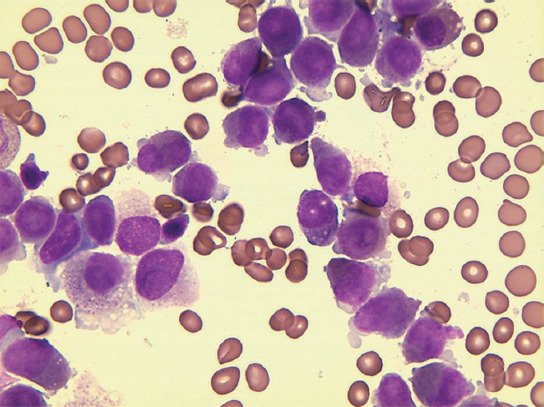
Bone marrow smear of the patient on admission.

A 62-year-old male presented with pharyngalgia, fatigue, and gum bleeding for 1 week. On admission, peripheral blood counts showed pancytopenia: Hemoglobin 78g/L platelet count 3×10^9^/L, leucocytes 2.53×10^9^/L with 75.6% neutrophils, and 15.3% lymphocytes. Abnormal coagulation screening tests showed prothrombin time 15.6s (normal 9.6–13.7s), activated partial thromboplastin time 25.1s (normal 20-40s), fibrinogen degradation products 168.98mg/L (normal <0-5 mg/L), and D-dimer > 20000 mg/L FEU (normal < 500mg/L FEU). The bone marrow (BM) was hypercellular and packed with 95.2% abnormal promyelocytes that were strongly positive for myeloperoxidase, leading to the morphological diagnosis of APL (Figure 1). The flow cytometry of BM specimens demonstrated that the blasts accounted for 87.27% which were positive for CD117, CD33, myeloperoxidase (MPO), CD13, CD58, CD38, and CD81. Based on the diagnosis and for the patient’s severely compromised overall condition, therapy with ATRA combined with ATO was initiated, followed by cytarabine (Ara-C) and daunorubicin (DNR)-based induction chemotherapy.

**FIGURE 2:**
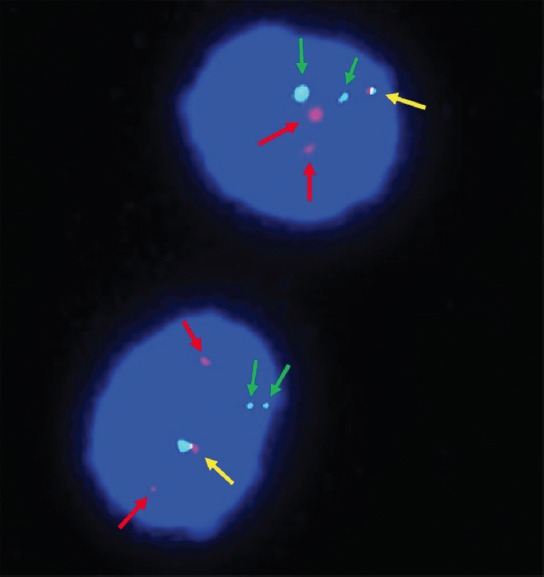
The fluorescence in situ hybridization analysis with painting probes for chromosomes 15 (green arrows) and 17 (red arrows). The fusion signals were marked by yellow arrows.

**FIGURE 3:**
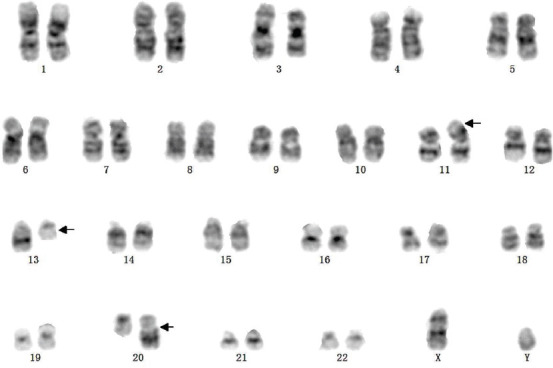
G-banded karyotype of the patient: 46,XY,add(11)(p15),?t(13;20)(q12;q11.2)[20]. The abnormal chromosomes are indicated by arrows.

Subsequently, the fluorescence in situ hybridization (FISH) analysis with a probe specific for the *PML* locus at 15q22 and the *RARα* locus at 17q21 was performed according to standard procedures. The atypical *PML/ RARα* fusion fluorescence signal, two red signals (*PML*), one green signal (*RARα*) and one yellow signal (*PML/RARα* fusion) or two red signals (*PML*), two green signals (*RARα*), and one yellow signal (2R1G1Y/2R2G1Y), was revealed in 91% (182/200) interphase cells (Figure 2). Quantitative real-time polymerase chain reaction (RT-qPCR) analysis showed that the major *PML/RARα* transcript harbored the three type breakpoints. The copy number of *PML/RARα* longtype was 19500, the varianttype was 11100, and the shorttype was 610. The longtype breakpoint cluster region is the main transcript in this patient, and the other breakpoints stem from alternative splicing events. The copy number of the internal reference gene, ABL1, was 157000. The relative expression of *PML/RARα* transcripts was 19%. However, the cytogenetic study showed an aberrant karyotype of a structural aberration in the short arm of chromosome 11 and translocation of chromosome 13 and chromosome 20, described as 46,XY,add(11)(p15), and ?t(13;20)(q12;q11.2)[20]/ (Figure 3) according to ISCN 1995 [9]. Furthermore, the next-generation sequencing (NGS) detected *FLT3*, *WT1*, and *KRAS* mutations (Table 1).

**TABLE 1:**
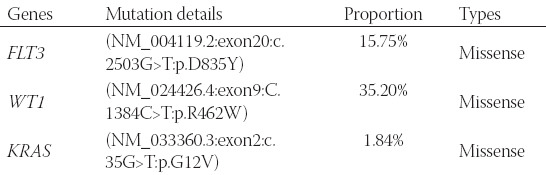
The Mutation types are detected by next-generation sequence

After 21 days, the patient obtained morphology complete remission (CR) with a significant reduction of myeloid promyelocytes from 95.2% to 0.4%. The blasts (CD34+ CD117+ CD33+ HLA-DR + CD38+) were accounted for 0.012% detected by flow cytometry, while there was no significant improvement in leucocytes or hemoglobin. FISH still can detect 80% atypical 2R1G1Y/2R2G1Y fusion fluorescence signal in interphase cells, and 23.1% *PML/RARα* transcripts were detected by RT-qPCR. Subsequently, the patient received the second cycle induction treatment with ATRA and ATO.

**FIGURE 4:**
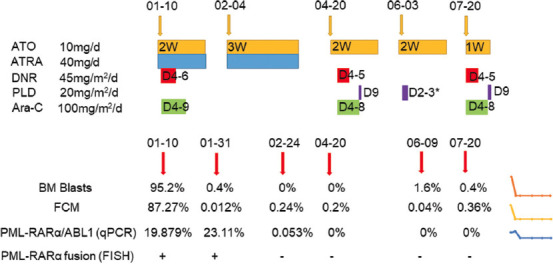
The treatment process of the patient. *D3 used half the drug dose. Aberration: ATO, arsenic trioxide; ATRA, all-trans retinoic acid; DNR, daunorubicin; PLD: pegylated liposomal doxorubicin; Ara-C: Cytarabine; FCM: Flow cytometer; qPCR: Quantitative polymerase chain reaction; FISH: Fluorescence in situ hybridization.

After the second treatment cycle, cytogenetic remission was still achieved. About 1% (2/200) 3R3G atypical fluorescence signal but no fusion signal was detected by FISH, 0.053% *PML/RARα* transcripts were detected by RT-qPCR, and 0.24% blasts (CD34-CD117+ CD56+ HLA-DR+CD38+) were detected by flow cytometry. The results of RT-qPCR and flow cytometry suggested minimal residual disease (MRD). NGS and CC analyses showed that the mutations and the karyotypes presented normal. After a recovery phase, a comprehensive review showed that there were no blasts or *PML/RARα* fusion genes in BM, which indicated a stable CR status. The patient then received post-remission therapy, including ATO, Ara-C, and DNR. The treatment process is shown in Figure 4, where the patient was still under the CR status.

## DISCUSSION

The clinical features of APL patients can be similar to those of other AML subtypes, and disseminated intravascular coagulation (DIC) and hyperfibrinolysis are the dominating complications directly affecting the clinical outcomes [10]. With ATRA and/or ATO, more than 90% of patients achieve long-term remission, and BM transplantation is limited to patients who relapse for the first time [11,12]. The classic t(15;17) leads to *PML* and *RARα* rearrangement at the molecular level [4]. About 9% of APL patients don’t have t(15;17) translocation, although some are still expressed *PML/RARα* fusion gene. These cases are believed to be caused by sub-microscopically inserted *PML/RARα* or more complex rearrangements, with hidden or masked transcripts, which could not be detected by the CC test. When karyotype fails to provide effective information, either due to a lack of metaphases or poor-quality metaphases or due to complex or cryptic rearrangements, the FISH analysis may help to verify the *PML/RARα* fusion and immediately initiate or maintain specific APL treatment. However, in some cases of APL, FISH does not detect the t(15;17) due to technical limitations, such as the probe size [5,13]. In these rare cases, the cryptic transcript is usually detected by RT-qPCR, and these patients can still benefit from ATRA and ATO since both are targeted therapies against the PML/RARa protein activity, although the patients are lacking the classic translocation.

Here, we reported a masked t(15;17) APL patient with a complex karyotype add(11)(p15) and t(13;20)(q12;q11.2). To the best of our knowledge, this is the first time this karyotype is reported in APL. Typical APL with *PML/RARα* and 1R1G2F signals are commonly detected by FISH, which indicates the classical t(15;17). In our patient, 182 out of 200 cells (91%) exhibited 2R2G1F or 2R1G1F signals which have also been reported in some cases with complex translocations such as three-way or four-way translocation [7,8]. Another indirect evidence was the 1% 3R3G atypical fluorescence signal that appeared at the third assessment. Although the signal did not reach the cutoff value of over 1%, considering the timepoint of CR, it still provides some information that the extra one red and one green signal represent chromosome 15 and 17, and that they may participate in translocation with other chromosomes. Normally, a three- or four-way translocation can be detected by CC, but in our case, we only detected a t(13;20)(q12;q11.2) and add(11)(p15) without t(15;17) which implied the submicroscopic insert. Kouichi Haraguchi et al. reported a case of masked t(15;17) with a *PML/RARα* insertion into chromosome 4 detected by spectral karyotyping (SKT) and FISH analyses [14]. Gudrun Göhring et al. reported a masked t(15;17) with a complex karyotype involving chromosomes 4, 6, 8, 10, 17, 18, and 21, a translocation between chromosomes 5 and 17 was detected by multi-color FISH (mFISH) analysis [15]. SKT and mFISH are both effectively applied to cases with complex karyotypes [16,17].

We also reviewed APL case reports that included the aberrant karyotypes of chromosome 13, 20, and 11, listed in Table 2. A complex four-way translocation has been reported, including chromosome 13 and chromosome 20, but the breakpoints were located at 13q22 and 20p13. This patient received ATRA treatment but soon died a week after admission [3]. As for the chromosome 13 band, 13q14 [18], 13q31 [19], and 13q12 [20] also have been reported as unusual karyotypes in APL patients. Paola Temperani et al. reported a case of APL patient whose t(12;13)(p13.2;q14) karyotype was detected not at the time of diagnosis but the second relapse[18]. Yuting Tang et al. described a case of a young APL patient with a masked del13(q31) who obtained CR 3 times but died soon after the third relapse due to sepsis [19]. Giorgina Specchia et al. described a secondary APL case following a renal transplant with complex karyotypes of +8,t(13;22)(q12;q13). The patient also achieved CR without extra hematologic toxicity during induction and consolidation treatment [20]. As for the chromosome 20 band, besides, 20q11.2[7], 20p13[3,22] and 20q12[21] have also been reported in APL patients. Zaida Garcı´a-Casado et al. reported a patient with t(17;20)(q21;q12) whose survival time was more than 5 years [21]. Another case with 20p13 was described that included a complex three-way translocation [22], but the outcome of the patient was favorable which was contrary to the patient with four-way translocation [3]. Jun Yamanouchi et al. described a case that also had a complex four-way translocation, including chromosome 20, the breakpoint was q11.2 which was the same as in our patient, but the clinical character of the karyotype remains to be explored [7]. As for the chromosome 11 band, 11q23 usually induces PLZF/RARa fusion protein [23], Trisomy 11 [24], and 11q13 [25-27] have also been reported. The role of 11p15 has been well described in non-M3 AML, usually participating in the karyotype of t(11;12)(p15;q13), which leads to the *NUP98-HOXC11*[28,29] or *NUP98-HOXC13*[30,31] fusion genes. Esperanza et al. reported an APL patient with t(11;12)(p15;q13) karyotype and unusual *NUP98/RARG* fusion genes, who achieved a favorable outcome with standard chemotherapy [32]. However, the role of add(11)(p15) in APL has not been explored. The three breakpoints have been reported before but the combination is a novel finding, which implies non-random events. As far as we know, the presence of complex karyotypes of our patient does not affect the therapeutic effect of chemotherapy combined with ATRA and ATO.

**TABLE 2:**
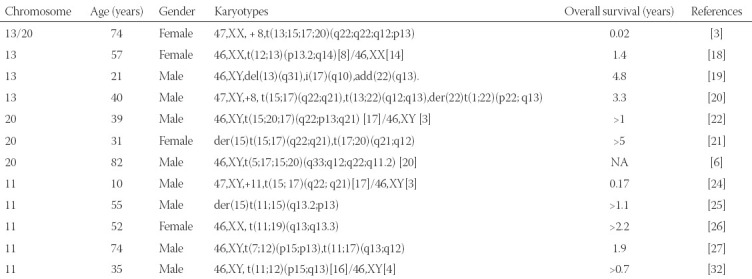
Cases of APL with aberrant chromosome 13, 20, and 11

In addition, *FLT3*, *KRAS*, and *WT1* mutations were detected by NGS in the present case. Based on the classical molecular landscape of 1540 AML patients, 4% (60/1540) of patients were classified with typical t(15;17)(q22;q12), and the most common mutants were *FLT3*-ITD and *WT1* that account for 35% and 17%, respectively, in APL cases [2]. *FLT3* encodes FMS-like tyrosine kinase 3, and the internal tandem duplication (ITD) is the most common aberration in APL [33]. *FLT3*-ITD induces constitutive activation of the tyrosine kinase receptor which may contribute to the pathogenesis and outcome of APL patients. About 8% of APL patients harbor another point mutation of *FLT3* in the codon for aspartic acid 835 (D835). Researches have reported that both two types of mutated *FLT3* are related to worse outcomes and high leukocyte counts [34,35]. The Wilms tumor 1 (*WT1*) gene encodes a protein that contains zinc-finger motifs, which plays an important role in hematopoiesis and elevated expression in AML as well as APL. The role of *WT1* in APL still needs to be explored, but a piece of indirect evidence showed that the *WT1* gene was the only mutated gene in relapsed patients and may influence the clinical outcomes [36]. *KRAS* is one of the three small GTP proteins of the RAS family which plays a crucial role in AML oncogenesis. However, the knowledge about the prevalence of *KRAS* mutation and the significance in APL is limited [37].

In general, almost all APL patients with complex translocations harboring *PML/RARα* fusion, including our case, have a good response to ATRA and ATO, similar to the typical patients with t(15;17). Our case may indicate that the translocation involving 13q12 and 20q11.2 and add(11)(p15) has no detrimental effect on the response to the ATRA and ATO. The patient achieved CR, and consolidation treatment will be followed up. However, considering the *FLT3* mutation and MRD which indicate poor clinical outcomes and relapse, medical observation must continue closely.

## CONCLUSION

We report a cryptic t(15;17) APL patient with a complex karyotype, including add(11)(p15) and t(13;20)(q12;q11.2), who achieved CR with polychemotherapy combined with ATRA and ATO targeted therapy. This is the first report of a cryptic APL contained complex karyotype involving chromosomes 11, 13, and 20, which seems not to alter the effectiveness of chemotherapy combined with ATRA and ATO.
